# A Case of Follicular Lymphoma Mimicking Idiopathic Retroperitoneal Fibrosis

**DOI:** 10.7759/cureus.32031

**Published:** 2022-11-29

**Authors:** Xiaoxi Lan, Ronghua Hu, Tianbin Song, Lu Gan, Wanling Sun

**Affiliations:** 1 Department of Hematology, Xuanwu Hospital, Capital Medical University, Beijing, CHN; 2 Department of Nuclear Medicine, Xuanwu Hospital, Capital Medical University, Beijing, CHN; 3 Department of Pathology, Bazhou People's Hospital, Korla, CHN

**Keywords:** malignant tumors, computed tomography, positron emission tomography, retroperitoneal fibrosis, lymphoma

## Abstract

Retroperitoneal fibrosis (RF) is a rare disease, which can be primary (idiopathic) or secondary. We present the case of a 56-year-old patient with symptomatic RF, in whom, after ineffective treatment with glucocorticoids, immunosuppressants, and non-steroidal anti-inflammatory drugs for one year and a progressive clinical course, a follicular lymphoma in the retroperitoneal space and several lymphoma nodes was identified. We also include a literature review on differential diagnosis through image inspection and case reports of lymphoma mimicking RF.

## Introduction

Retroperitoneal fibrosis (RF), a rare disease characterized by progressive inflammatory, fibrous mass in the retroperitoneal space, was first described in 1905. Nowadays, more than a hundred years later, the diagnosis of this disease is not difficult, but due to the variety of etiologies and the unclear pathogenesis, further differential diagnosis is very important for RF patients, especially the need to determine the malignancy of retroperitoneal mass. It may require repeated judgments throughout the whole course of the disease.

## Case presentation

**Figure 1:**
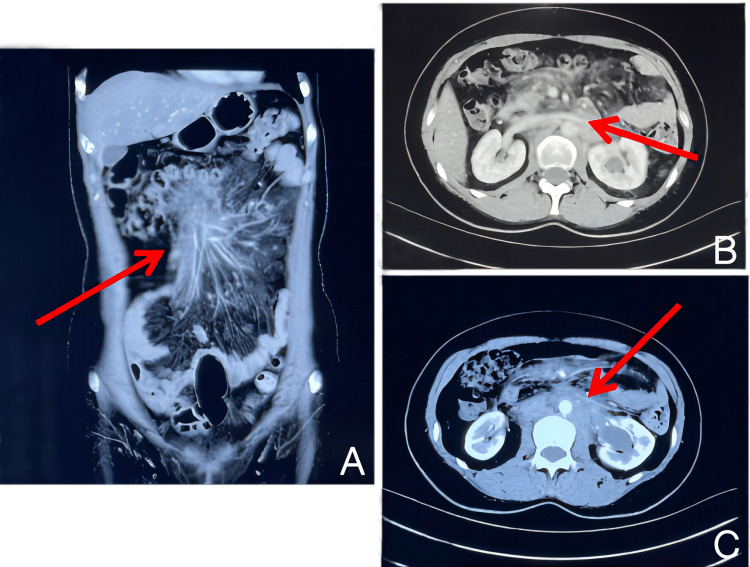
Abdominal CT of March, 2017. Soft tissue in the retroperitoneum, which invaded the small intestine (A) and, surrounding the bilateral ureters, caused bilateral hydronephrosis (B,C).

A 56-year-old woman with headache, back pain, nausea, and new-onset hypertension presented to the emergency in January 2016. She did not have a remarkable medical and family history. Blood testing revealed normal blood cells and renal, pancreatic, and liver functions. The kidney ultrasound revealed a dilation of the left renal pelvis. Then contrast-enhanced computed tomography (CT) scan of the abdomen and pelvis showed soft tissue in the retroperitoneum, which invaded the nearby small intestine and surrounded the bilateral ureters, and caused bilateral hydronephrosis. These findings were suggestive of retroperitoneal fibrosis. She was admitted to rheumatology. The rheumatologist did not find any other possible etiologic factors except a positive anti-SSA antibody, so she was diagnosed with idiopathic RF (IRF). She received treatment with methyl prednisone 32mg per day, tamoxifen 10mg twice a day, and methotrexate 10mg per week for one year. However, a repeat CT scan of the abdomen and pelvis in March 2017 revealed no changes（Figure 1).

**Figure 2:**
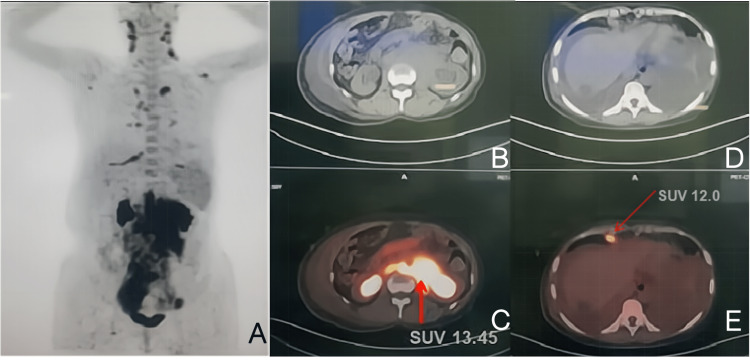
PET-CT of December 2018. The soft tissue with FDG hypermetabolism located in the nearby abdominal aorta, vena cava, and small intestine mesenteric and presacral area (A-E). The SUVmax of it was 13.45 (C). PET: positron emission tomography; FDG: fluorodeoxyglucose; SUVmax: maximum standardized uptake value

In December 2018, the patient went to the hospital because of bloating, coughing, and wheezing for one month. A positron emission tomography (PET)/CT scan showed that the soft tissue with fluorodeoxyglucose (FDG) hypermetabolism was located in the nearby abdominal aorta, vena cava, and small intestine mesenteric and presacral area. The maximum standardized uptake value (SUVmax) of it was 13.45（Figure 2). The boundary between the lesion and the left kidney and bilateral psoas major muscle was not clear. It grew forward and downward along the bilateral external iliac arteries. Multiple enlarged lymph nodes with FDG hypermetabolism were found in the bilateral neck, bilateral internal mammary area, mediastinum, bilateral hilar area, bilateral costophrenic angle, and right groin regions. There was a large amount of fluid effusion in the abdominal and thoracic cavities.

**Figure 3:**
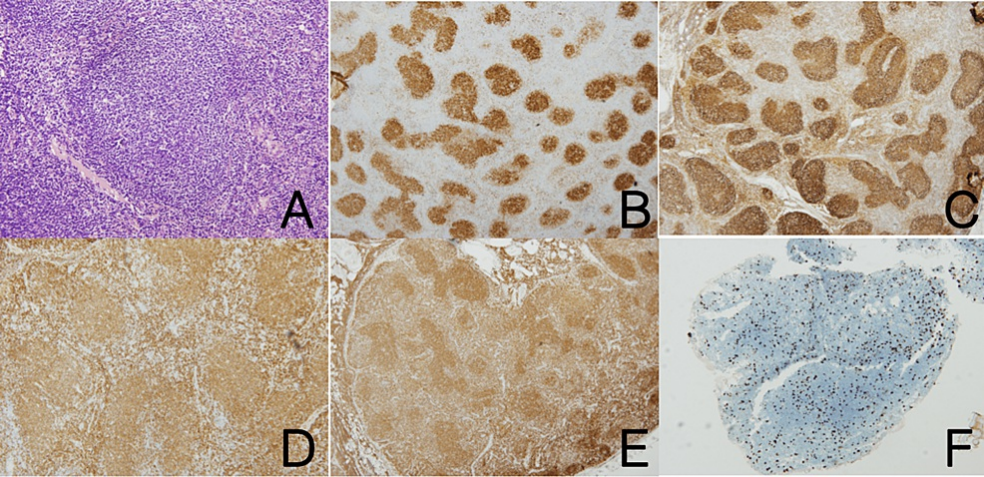
Immunohistochemical features: (A) pathological image of lymph node without normal lymphoid follicles using H&E staining (original magnification ×200); Immunohistochemical features show medium-sized lymphocytes were positive for CD20 (C), CD10 (B), Bcl-2 (E), Bcl-6 (D), and 15% positive for Ki67 (F) H&E: hematoxylin and eosin

She underwent a biopsy of a cervical lymph node and a puncture of the lesion in the retroperitoneum region. The pathology results demonstrated the lesion was follicular lymphoma (grade 1-2). The immunohistochemical results revealed a lymphoid infiltrate composed predominantly of CD20-positive medium-size B lymphocytes that were CD10, BCL2, BCL6 positive, and Ki67 15%-positive, without normal lymphoid follicles (Figure 3).

She received six cycles of R-CHOP (rituximab-cyclophosphamide-hydroxydaunorubicin-Oncovin-prednisone)-28 chemotherapy (rituximab at 375 mg/m^2^/day 0, vindesine 4mg/day 1, epirubicin 75mg/m^2^/day 1, prednisone 100mg/day 1-5, cyclophosphamide 0.75g/day 1). She obtained complete remission after six cycles of chemotherapy. In addition, she received rituximab therapy every two months at 375 mg/m^2^ plus lenalidomide at 25mg/day for two years after the chemotherapy. The last follow-up was in February 2022, and she remained in complete remission status.

## Discussion

RF is a rare disease that can occur at any age. The incidence rate is only one in a million. RF may be idiopathic or secondary, and benign or malignant, as a fibrous, inflammatory soft tissue that stretches in the retroperitoneum, encompassing the aorta and vena cava, and extending along the iliac vessels to the pelvis. The fibrosis often progresses later to encompass the ureters and be responsible for ureterohydronephrosis. Reliable classification of RF is often not possible. Peisen et al. concluded that RF may be a consequence of, or a coexisting condition with, arterial vascular disease, an autoimmune disease, vasculitis, IgG4-associated RF based on IgG4-positive plasma cell infiltrates, drug-induced RF, RF resulting from radiation or surgery, and malignant RF [1]. In 75% of cases, as the etiology cannot be determined, it is diagnosed as idiopathic retroperitoneal fibrosis (IRF) [2]. There is no difference in imaging findings of benign and malignant RF. The biopsy with pathological examination remains the only means of establishing the diagnosis of malignancy [3]. 

In our case, the patient first went to the hospital because of headache, back pain, nausea, and new-onset hypertension, and the CT examination showed a mass in the retroperitoneum that surrounded the adjacent iliac vessels and the urinary tract. It demonstrated a diagnosis of RF. The rheumatologist, who found the patient’s morphological manifestation consistent with IRF, diagnosed her after a complete differentiation except for a biopsy of the lesion, and treated her with methyl prednisone, tamoxifen, and methotrexate. Indeed, some authors presented criteria for a malignant cause of RF by morphological examinations [4]. In a retrospective review by Zhang et al., which included 19 newly diagnosed benign RF patients and 23 patients with lymphoma involving retroperitoneum, homogeneous enhancement, and pelvic extension were significantly more common in benign RF, while the involvement of additional nodes, suprarenal extension, and aortic displacement was significantly more common in lymphoma [5]. And lesion size at the para‑aorta was significantly greater in lymphoma. What’s more, in a study of PET-CT among patients of idiopathic IRF, lymphoma, and retroperitoneal metastatic malignancy, the dimension of retroperitoneal lesions in IRF were smaller than those in retroperitoneal lymphoma, and IRF displayed a lower SUVmax than lymphoma and metastatic malignancy [6]. Lymph nodes located in maxillary, retroperitoneal, supraclavicular, inguinal, or peritoneal sites were less frequently observed in IRF. However, these morphological and functional characteristics of lymphoma did not all happen at the beginning of the disease, such as in our patient whose soft tissue mass enlarged after about three years after the first diagnosis.

Finally, we searched eight cases reports of lymphoma mimicking RF in PubMed, which included two cases of B-cell Non-Hodgkin lymphoma [7,8], three cases of follicular lymphoma [9-11], one case of diffuse large B cell lymphoma [12], and two cases of anaplastic large cell lymphoma [13,14]. The results of such diverse pathological types seem to suggest that lymphoma does not develop from RF, so it is necessary for all newly diagnosed patients with RF to undergo lesion biopsy to exclude malignant diseases.

## Conclusions

In addition to the common inducing factors, we should be more alert to RF secondary to malignant tumors, such as lymphoma. With the development of imaging technology, PET-CT examination may important for the early detection of malignant tumors. It is important for early diagnosis and prognosis to obtain histopathology by puncture or surgery at the same time.
